# A Tale of Two Recent Spills—Comparison of 2014 Galveston Bay and 2010 Deepwater Horizon Oil Spill Residues

**DOI:** 10.1371/journal.pone.0118098

**Published:** 2015-02-25

**Authors:** Fang Yin, Joel S. Hayworth, T. Prabhakar Clement

**Affiliations:** Environmental Engineering Program, Department of Civil Engineering, Auburn University, Auburn, Alabama, United States of America; Northwest Fisheries Science Center, NOAA Fisheries, UNITED STATES

## Abstract

Managing oil spill residues washing onto sandy beaches is a common worldwide environmental problem. In this study, we have analyzed the first-arrival oil spill residues collected from two Gulf of Mexico (GOM) beach systems following two recent oil spills: the 2014 Galveston Bay (GB) oil spill, and the 2010 *Deepwater Horizon* (DWH) oil spill. This is the first study to provide field observations and chemical characterization data for the 2014 GB oil spill. Here we compare the physical and chemical characteristics of GB oil spill samples with DWH oil spill samples and present their similarities and differences. Our field observations indicate that both oil spills had similar shoreline deposition patterns; however, their physical and chemical characteristics differed considerably. We highlight these differences, discuss their implications, and interpret GB data in light of lessons learned from previously published DWH oil spill studies. These analyses are further used to assess the long-term fate of GB oil spill residues and their potential environmental impacts.

## Introduction

On March 22, 2014, on the weekend of the 25th anniversary of the catastrophic *Exxon Valdez* oil spill in Alaska, the bulk carrier *M/V Summer Wind* collided with the oil barge *Kirby*, near Texas City, about 50 km southeast of Houston, Texas. The accident released approximately 168,000 gallons of marine fuel oil (known as RMG-380, a highly viscous, sticky, heavy black oil) into Galveston Bay (GB). After the accident, oil residues began washing up on several beaches along GB. The oil spill also spread into the Gulf of Mexico (GOM) and within a week oil was rapidly transported by shoreline currents to the Matagorda Island Wildlife Management region, located about 200 km south of GB. By the end of March, overflight observers noted beached oil being rapidly buried under clean sand near Matagorda Island [1]. Unfortunately, oil spill incidents like these occur in GB on a regular basis: according to the Texas General Land Office, 3,954 oil spills occurred in GB between 1998 and 2010 [2].

Oil spill residues washing onto shores is a common problem for many northern GOM beach systems. In 2010, the *Deepwater Horizon* (DWH) accident released about 210 million gallons of Louisiana light sweet crude oil into the GOM impacting over 1,600 miles of shoreline, and depositing oil on Florida, Alabama, Mississippi, Louisiana and Texas beaches. Negative environmental, ecological, social, and economic consequences of this event continue today [3–8]. Impacted beaches and dunes, estuaries, and tidal brackish and freshwater wetlands and the numerous species inhabiting them were, and remain, at risk of long-term detrimental effects as a result the spill [3–6,9,10].

The amount of oil released during the GB accident was relatively small compared to the DWH accident. The physicochemical characteristics of these two oils are also different. Fuel oil released during the GB spill was a heavy, viscous, refined fluid containing low levels of volatile hydrocarbons, while oil released during the DWH accident (MC252 crude oil) was an unrefined, low viscosity, sweet crude enriched in light, volatile hydrocarbons. Another major difference between the two events is that the GB spill was a surface release discharged about a kilometer away from the nearest shoreline; while the DWH event occurred about 75 km from the nearest shoreline, about 1.5 km under water. Uniquely, large volumes of chemical dispersants were also injected subsurface during the DWH spill response. Owing to its proximity to the shoreline, GB oil weathered in marine waters for only a few hours to days before being deposited on nearby beaches. Also, the GB spill occurred close to several sensitive wildlife areas during breeding season of migratory birds and marine species. DWH oil, on the other hand, was weathered by ocean-scale processes such as volatilization, dissolution, emulsification, photo-degradation and/or biodegradation for 3 or more weeks before being deposited on northern GOM shorelines. Table 1 summarizes some of the key features of these two oil spills.

**Table 1:** Comparisons of Galveston Bay and Deepwater Horizon oil spills.

	Galveston Bay Oil Spill	Deepwater Horizon Oil Spill
API at 15°C	∼ 11 [43]	∼ 35 [44]
Viscosity (cSt)	∼ 380 at 50^°^C [43]	∼5 at 40^°^C [45]
Volume of the spill	∼168,000 gallons	∼210 million gallons
Type of oil	Refined marine fuel oil	Unrefined Louisiana sweet crude oil
Type of accident	Vessels collision	Explosion of oil rig
Type of spill	Tanker release	Well head release
Point of release	Surface oil spill	Subsurface oil spill, ∼1.5 km below sea
Spill location	∼ 1 km away from beaches	∼70 km away from beaches
Weathering patterns	Fate of remnant oil is yet to be studied	Fate of remnant oil has been studied for ∼4 years [7,8,10,15,16,19,38,46,47]

The objective of this study is to compare observational and chemical characterization data of first-arrival oil samples collected from GB and DWH spill-impacted beaches. Chemical characterization efforts included the measurement of concentrations of n-alkanes, several biomarkers, five groups of alkylated polycyclic aromatic hydrocarbons (PAHs) and seventeen other PAHs. Biomarker data for the GB oil presented in this study are important since they can be used for identifying and differentiating GB residue from oil residues from other sources, and are also useful for understanding weathering levels. PAH data are useful for comparing and quantifying potential long-term environmental impacts of GB and DWH oil spill residues.

## Materials and Methods

Organic solvents used in this study were of analytical or higher grade and were purchased from VWR International (Suwanee, GA). Silica gel (60–200 μm) and anhydrous sodium sulfate (ACS grade) were also purchased from VWR International. Prior to use, silica gel was activated using well-established procedures [11]. C_8_-C_40_ alkanes, pristane and phytane mixtures and hexadecane-d_*34*_ were purchased from Sigma-Aldrich. Biomarkers, namely C_30_αβ-hopane (17α(*H*),21β(*H*)-hopane), C_27_ααα(R)-sterane (5α,14α,17α(*H*) cholestane 20R), and C_30_ββ-hopane (17β(*H*),21β(*H*)-hopane) were purchased form Chiron, Norway. The PAH reference standard consisting of 27 different PAHs (naphthalene, 1-methylnaphthalene, 2-methylnaphthalene, 2,6-dimethylnaphthalene, 2,3,5-trimethylnaphthalene, biphenyl, acenaphthylene, acenaphthene, fluorene, phenanthrene, 1-methylphenanthrene, anthracene, dibenzothiophene, fluoranthene, pyrene, benz[*a*]anthracene, chrysene, benzo[*b*]fluoranthene, benzo[*j*]fluoranthene, benzo[*k*]fluoranthene, benzo[*e*]pyrene, benzo[*a*]pyrene, perylene, dibenz[*a*,*c*]anthracene, dibenz[*a*,*h*]anthracene, indeno[1,2,3,-*cd*]pyrene and benzo[*ghi*]perylene) was purchased from Agilent (Wilmington, DE). The reference solution for *p*-terphenyl-d_*14*_ was purchased from AccuStandard (New Haven, CT).

The oil spill samples recovered from GB beaches contained sand and other inorganics, and the organic fraction in the sample was estimated to be 65% (*w*/*w*) using a standard dichloromethane extraction procedure [12]. The DWH oil spill sample was free of sand and other residues and it fully dissolved in dichloromethane. For biomarker and PAH quantitative assessments, about 20 mg of GB or DWH sample was dissolved in hexane and prepared using a column chromatographic fractionation method [10]. The hexane fraction (F1) was used for n-alkanes and biomarker analysis, and the hexane:dichloromethane (50%, v/v) fraction (F2) was used for PAH analysis. Each sample was prepared in duplicate and analyzed in triplicate.

Both F1 and F2 elutes were analyzed using an Agilent 7890 GC equipped with Agilent 7000B QqQ mass spectrometer detector. Single ion monitoring (SIM) mode was used for F1 analysis with a m/z value of 57 for n-alkanes [13], m/z of 78 for hexadecane-d_*34*_, m/z of 191 for hopanes and m/z of 217 for steranes [12]. The five groups of alkylated-PAH homologs and seventeen other PAHs in the F2 fraction were analyzed using SIM and multiple reaction monitoring (MRM) methods, respectively, following previously established analytical approaches [10,14].

Quantification of n-alkanes was achieved by integrating all major chromatographic peaks of n-alkanes observed at the target ion m/z of 57. Hexadecane-d_*34*_ was used as the internal standard. The total concentrations of hopanes and steranes were quantified by integrating appropriate peak areas of chromatograms observed at m/z 191 (retention time from 37 to 46 minutes) and m/z 217 (retention time from 32 to 40 minutes), respectively. The reference standards used for quantification were C_30_αβ-hopane for hopanes, and C_27_ααα(R)-sterane for steranes. C_30_ββ-hopane was used as an internal standard to normalize the response factors used for estimating total hopanes and steranes. Based on available alkylated PAH standards, five groups of alkylated PAHs were quantified in this study using previously developed methods [10,11]. The analytical standards used for quantifying various PAHs within these five groups were as follows: in Group-1, naphthalene was used for quantifying C_0_-naphthalene; 2-methylnaphthalene for C_1_-naphthalenes; 2,6-dimethylnaphthalene for C_2_-naphthalenes; and 2,3,5-trimethylnaphthalene for C_3_- and C_4_-naphthalenes. In Group-2, phenanthrene was used for C_0_-phenanthrene, and 1-methylphenanthrene for C_1_- to C_4_-phenanthrenes. In Group-3, dibenzothiophene was used for C_0_- to C_3_-dibenzothiophenes. In Group-4, fluorene was used for C_0_- to C_3_-fluorenes. In Group-5, chrysene was used for C_0_- to C_4_-chrysenes. Seventeen other PAHs were also quantified in this study, which included biphenyl, acenaphthylene, acenaphthene, anthracene, fluoranthene, pyrene, benz[*a*]anthracene, benzo[*b*]fluoranthene, benzo[*j*]fluoranthene, benzo[*k*]fluoranthene, benzo[*e*]pyrene, benzo[*a*]pyrene, perylene, dibenz[*a*,*c*]anthracene, dibenz[*a*,*h*]anthracene, indeno[1,2,3,-*cd*]pyrene and benzo[*ghi*]perylene. These compounds were quantified using the 27-PAH Agilent standard and previously published analytical procedures [10,15]. The internal standard *p*-terphenyl-d_*14*_ was used to normalize all PAH response factors.

## Field Observations and Samples Collection

**Fig 1:**
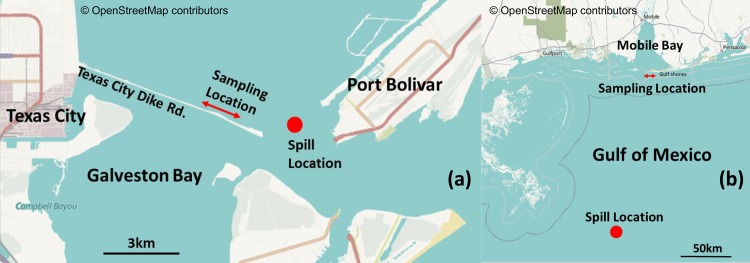
Locations of the two oil spills and sampling points: a) Galveston Bay spill; and b) Deepwater Horizon spill (maps from OpenStreetMap).

**Fig 2:**
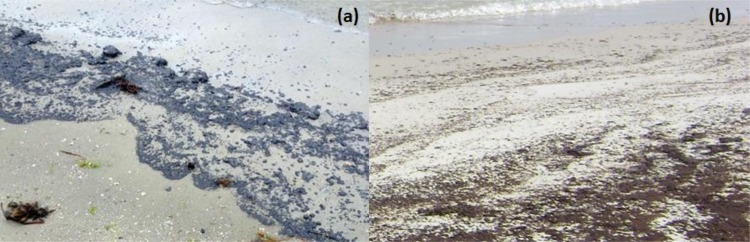
Comparison of Galveston Bay and Deepwater Horizon oil spill deposition patterns: a) blackish oily material deposited on a sandy beach in Galveston Bay, Texas (Photo taken on March 23^rd^, 2014, by NOAA's Office of Response and Restoration); b) brownish emulsified oil deposited on a sandy beach in Orange Beach, Alabama (Photo taken on June 11^th^, 2010, by Auburn University team).

**Fig 3:**
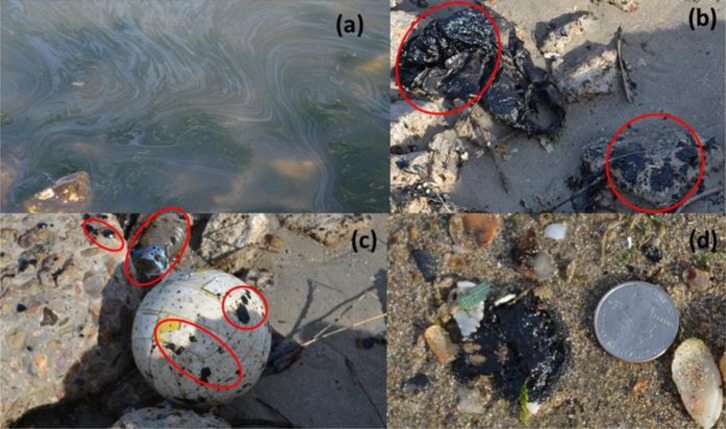
Field observation made at the Texas Dike road (Photographs taken on March 29^th^ 2014, by Auburn University team): a) oil sheen observed in nearshore water; b) oil on a plastic sheet and rocks; c) oil on rocks and on a beached soccer ball and other objects; and d) beached oil blobs observed close to the waterline.

Fig. 1 shows the GB and DWH oil spill sites and our sampling locations. No specific permissions were required for sampling at these locations. Also, the field studies did not involve endangered or protected species. GPS coordinates for our DWH field site in Alabama are: 30°16'42.8"N 87°33'17.1"W; GPS coordinates for our GB field site in Texas are: 29°22'22.6"N 94°49'48.6"W. GB oil began washing on GB beaches within few hours after the spill on March 22, 2014. The GB samples analyzed in this study were collected on March 29, 2014, from an amenity beach located along the Texas City Dike road, about 2 km away from the spill location. The DWH oil first arrived on Alabama’s beaches in early June, 2010, about a month after the accident, and the samples were collected on June 11, 2010 from Orange Beach, Alabama, located about 175 km from oil release location. Further details on the DWH field site, observed contamination patterns, and field sampling methods are discussed in Hayworth et al. [8] and Mulabagal et al. [12]. Fig. 2 shows typical first-arrival oil deposition patterns observed at these two field sites. Although the overall deposition patterns appear similar, the physical characteristics of oil residues were distinctly different. The GB first-arrival oil was black/grayish, highly viscous material, while the DWH first-arrival oil was a brownish, low viscosity emulsion. On the day of sampling (June 11, 2010), DWH oil was actively washing ashore along most of Alabama’s 50 km sandy beach system and public access to these contaminated beaches was unrestricted. In contrast, on the day of GB oil sampling (March 29, 2014) oil was washing ashore only along a limited stretch of GB shoreline, and public access to these active deposition areas was restricted. Our GB oil spill sampling efforts were completed near a sparsely contaminated area, located about 2 km from the spill site, which had previously been cleaned and reopened for public use. Fig. 3 shows the field observations made at this site. Despite active clean-up efforts, the shoreline water along these “cleaned areas” had a strong petroleum odor, and the nearshore water had patches of floating oil sheen (see Fig. 3A). We also observed oil adhering to rocks, beached objects and vegetation (Fig. 3B & C). Furthermore, small blobs of oil (about 2 cm diameter; see Fig. 3D) were randomly scattered in the intertidal zone. During our sampling effort, we collected oil adhered to rocks and beached objects and also collected several beached oil blobs from the intertidal zone. These samples were shipped to our laboratory for chemical analysis.

## Results and Discussion

### Chemical Characterization Data for n-Alkanes

**Fig 4:**
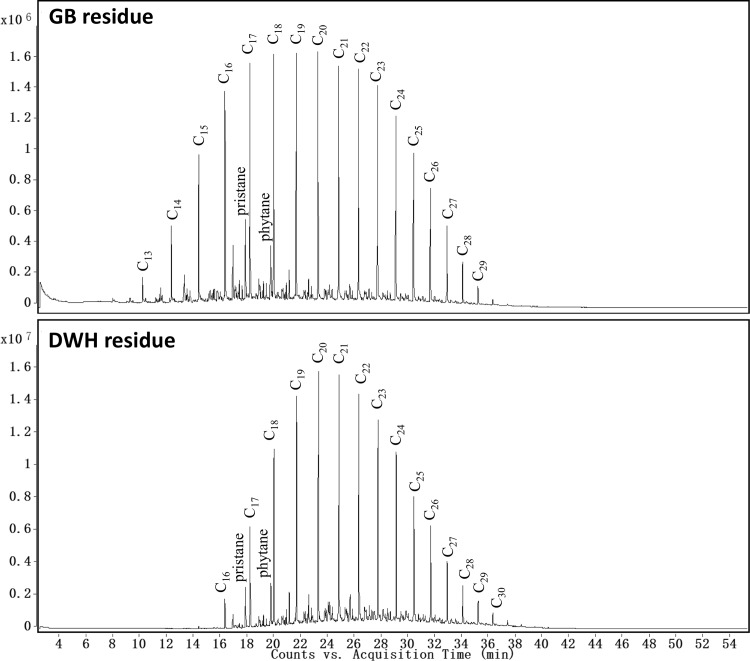
Comparison of extracted ion chromatograms of n-alkanes (m/z of 57) for Galveston Bay and Deepwater Horizon oil spill residues.

Fig. 4 shows the n-alkane chromatograms (m/z 57) of GB and DWH oil spill residues. The chromatogram for GB residues indicates the presence of n-alkane compounds ranging from C_13_ to C_29_. In comparison, the n-alkane profile for DWH oil residue was relatively narrow indicating the presence of compounds ranging from C_16_ to C_30_, and the lighter alkanes were absent in this sample. From literature data we know that unweathered DWH crude oil contained a wide range of n-alkanes starting from C_9_ [16]. Therefore, absence of light n-alkanes (i.e., compounds below C_16_) in the DWH first-arrival sample is due to ocean-scale weathering effects. The DWH samples were recovered about 50 days after the accident. During this period, the oil traveled over 175 km in marine waters with ocean-scale weathering processes selectively depleting most of the light n-alkanes. In contrast, the GB samples were recovered seven days post-accident; the oil traveled only about 2–3 km and experienced very little natural weathering; hence, the relative distribution of light n-alkanes are expected to be high in these samples. Also, both residues were collected during a similar season (around spring) from beaches with similar water temperatures. Thus, residence time in the marine environment is likely the primary driver for oil evaporation, with temperature playing a minor role [17].

**Fig 5:**
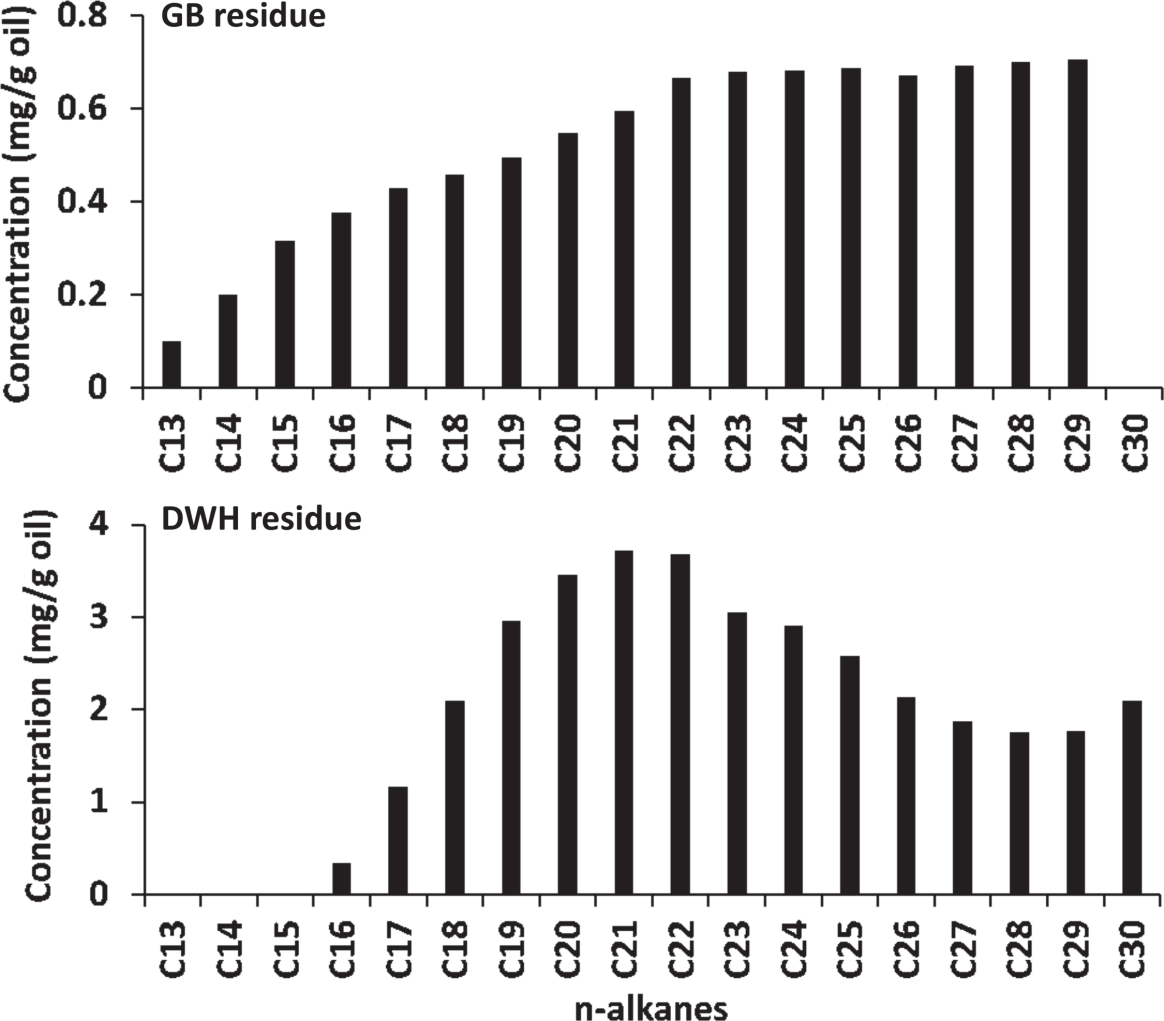
Concentration levels of various n-alkanes (ranging from C_13_ to C_30_) measured in Galveston Bay and Deepwater Horizon oil spill residues.

We also quantified n-alkane concentrations by integrating all major peaks for m/z 57, and the concentration levels for various n-alkanes ranging from C_13_ to C_30_ are presented in Fig. 5. Using the data shown in Fig. 5, the total amount (values reported as mean ± SD) of n-alkanes in GB and DWH samples are estimated to be 9 ± 1 and 37 ± 2 mg/g of oil, respectively. The total concentration of n-alkanes in GB residues is low since it is a highly refined fuel oil. The ratios of pristane/phytane, C_17_/pristane, and C_18_/phytane are often used for source identification [14]. Based on peak responses, the ratios of pristane/phytane, C_17_/pristane and C_18_/phytane were calculated as: 1.48 ± 0.04, 2.13 ± 0.04, and 3.21 ± 0.08 for GB sample, and 0.91 ± 0.01, 1.73 ± 0.01, and 2.84 ± 0.02 for DWH sample. These ratios are indicative of the differences in chemical characteristics of these two oils.

### Chemical Characterization Data for Biomarker Compounds

**Fig 6:**
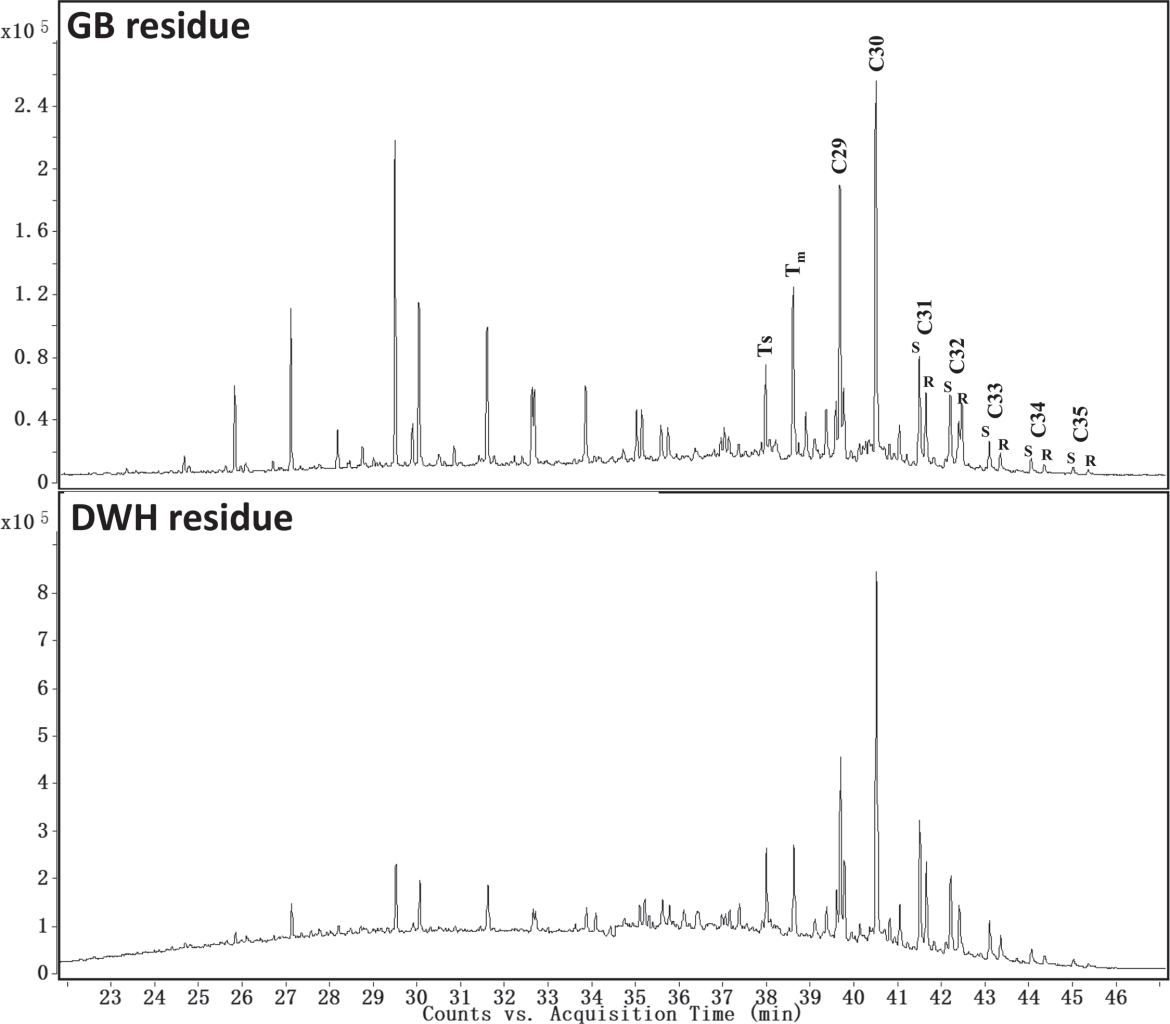
Comparison of extracted ion chromatograms of hopanes (m/z of 191) for Galveston Bay and Deepwater Horizon oil spill residues.

In this study we focused on the biomarker fingerprints of hopanes and steranes, which are the most widely used compounds for fingerprinting oil spill accidents [14,18]. Recently, Aeppli et al. [19] compared the fate of biomarkers in DWH oil spill residues and concluded that hopanes and steranes, quantified at m/z values of 191 and 217, respectively, are the most reliable signatures for fingerprinting DWH oil spill residues. Fig. 6 shows GC/MS chromatograms of hopanes (at m/z 191) present in GB and DWH residues. The total amount of hopanes in GB and DWH samples were estimated to be 380±30 mg/kg oil and 440±20 mg/kg oil, respectively. Analysis of hopane chromatograms show that in the DWH sample, hopane distribution ranged from C_27_ to C_35_ with C_30_αβ-hopane being the most abundant compound. The GB chromatogram, on the other hand, showed higher abundance of C_29_αβ and C_30_αβ hopanes; also, the response levels are higher for several other possible tricyclic or tetracyclic terpanes, yielding a wider fingerprint (see Fig. 6). It is well established that C_30_αβ-hopane is highly resistant to environmental weathering [14,19,20]; thus, the amount of C_30_αβ-hopane will increase over time, and this effect can be used to estimate the degree of weathering [12]. Furthermore, C_30_αβ-hopane can also be used as a recalcitrant internal biomarker for quantifying the degradation rates of other chemical compounds [20]. In this study, we estimated the concentrations of C_30_αβ-hopane in the GB residue as 81±6 mg/kg oil. The concentration of C30αβ-hopane in the DWH residue has already been reported in Mulabagal et al. [12] as 91±6 mg/kg oil. These concentration levels can be used as a starting point for understanding future weathering patterns of these oil residues.

**Fig 7:**
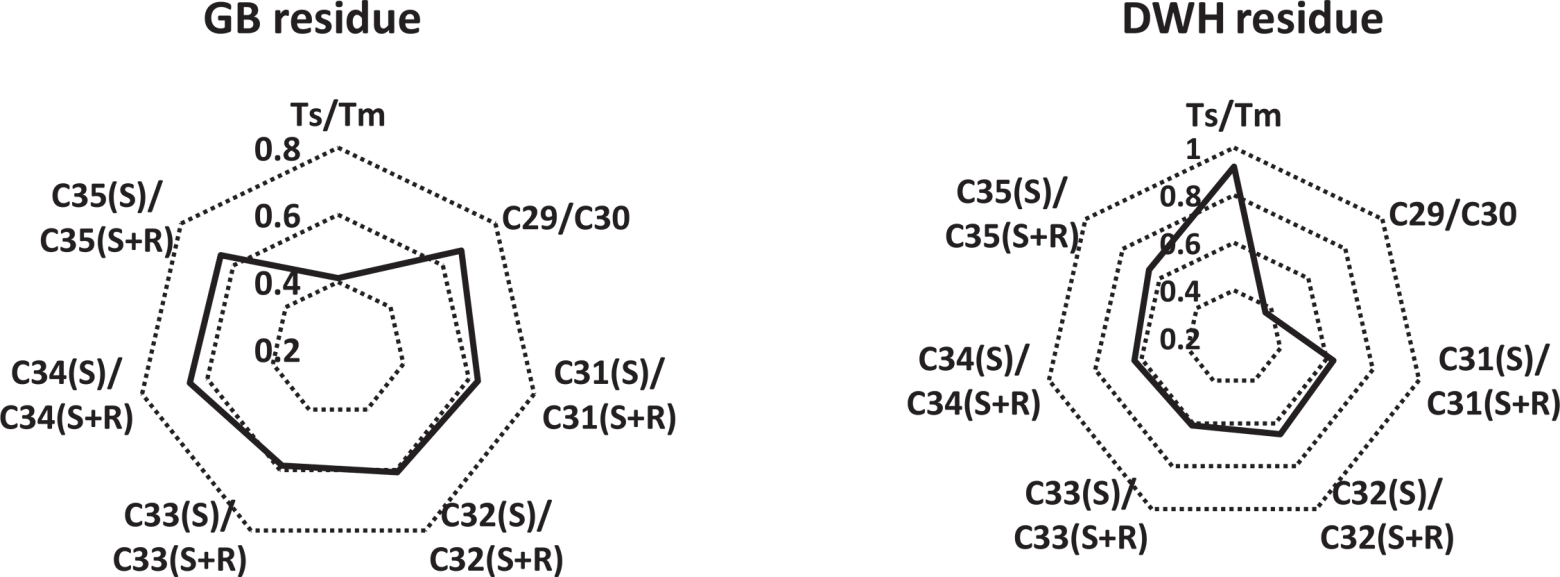
Radar plots of hopane diagnostic ratios of Galveston Bay and Deepwater Horizon oil spill residues.

The diagnostic ratios of different types of hopanes can be used to identify and differentiate oil spill sources [12,14,18,21]. Various ratios including those of T_s_/T_m_, C_29_/C_30_, C_31_(S)/C_31_(S+R), C_32_(S)/C_32_(S+R), C_33_(S)/C_33_(S+R), C_34_(S)/C_34_(S+R) and C_35_(S)/C_35_(S+R) in GB and DWH residues are summarized in Table 2 (a portion of the hopane data for DWH oil are from our previous work [12]). These data are also presented as radar plots in Fig. 7; the plots reveal that unique fingerprint patterns exist for these two oils, and these patterns can be used to differentiate these two spills from other past or future oil spills.

**Table 2:** Hopane and sterane diagnostic ratios (mean ± SD) estimated for Galveston Bay and Deepwater Horizon oil spill residues.

Diagnostic ratio	GB residue	DWH residue
Hopane ratio		
T_s_/T_m_	0.41±0.02	0.92±0.05
C_29_/C_30_	0.67±0.03	0.37±0.01
C_31_(S)/C_31_(S+R)	0.63±0.01	0.63±0.01
C_32_(S)/C_32_(S+R)	0.61±0.02	0.65±0.01
C_33_(S)/C_33_(S+R)	0.59±0.02	0.61±0.01
C_34_(S)/C_34_(S+R)	0.65±0.02	0.63±0.01
C_35_(S)/C_35_(S+R)	0.65±0.04	0.65±0.03
Sterane ratio		
DiaC_27_βα(S)/ DiaC_27_βα(R)	1.48±0.06	1.47±0.01
C_27_αββ(R+S)/C_29_αββ(R+S)	1.60±0.07	3.0±0.3
C_27_αββ(R+S)/C_27_(αββ(R+S)+ ααα(S+R))	0.50±0.01	0.67±0.01
C_28_αββ(R+S)/C_28_(αββ(R+S)+ ααα(S+R))	0.55±0.03	0.62±0.03
C_29_αββ(R+S)/C_29_(αββ(R+S)+ ααα(S+R))	0.38±0.02	0.51±0.01

**Fig 8:**
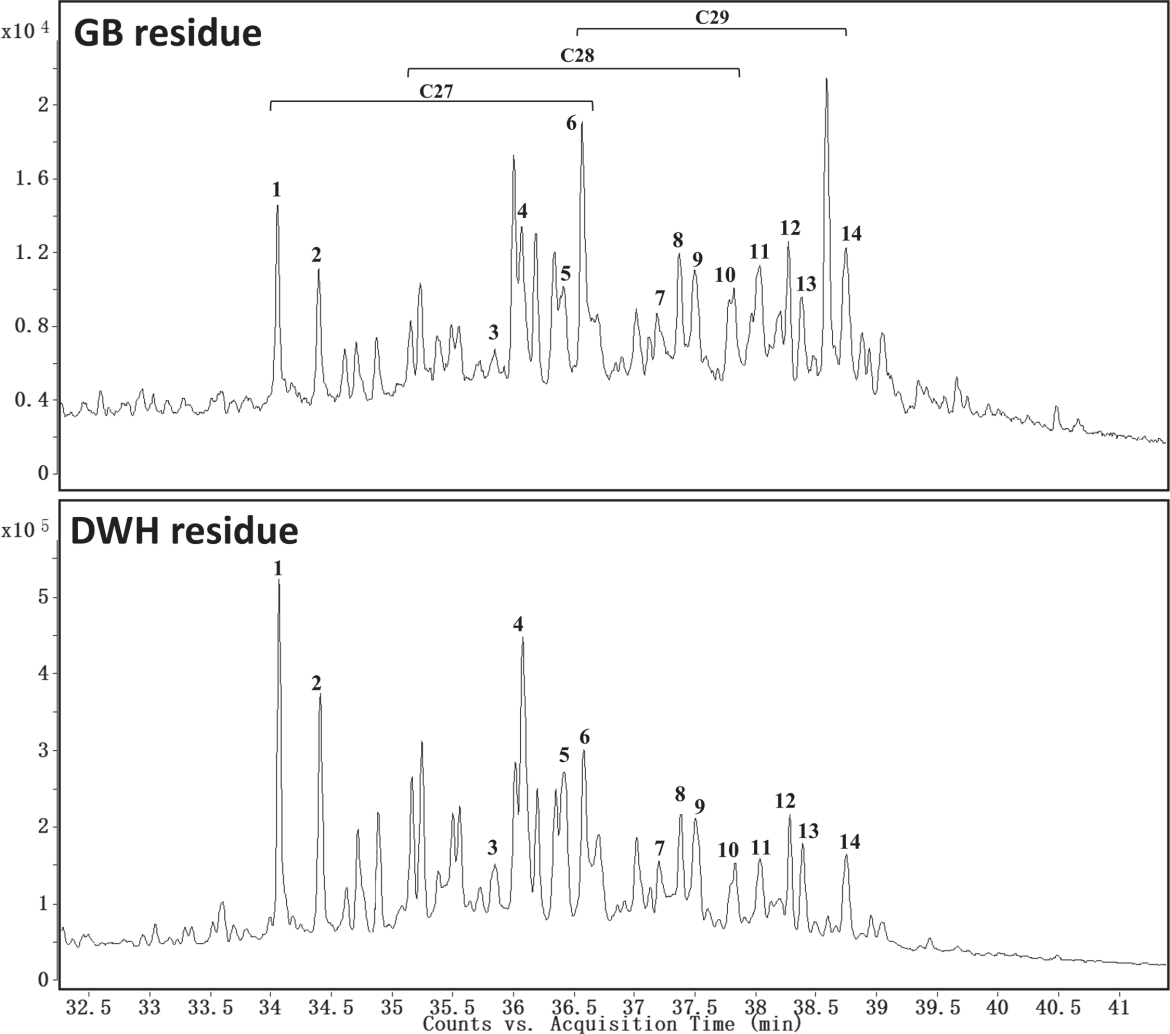
Comparison of extracted ion chromatograms of steranes (m/z of 217) for Galveston Bay and Deepwater Horizon oil spill residues [Peak 1: DiaC_27_βα(S); Peak 2: DiaC_27_βα(R); Peak 3: C_27_ααα(S); Peak 4: C_27_αββ(R); Peak 5: C_27_αββ(S); Peak 6: C_27_ααα(R); Peak 7: C_28_ααα(S); Peak 8: C_28_αββ(R); Peak 9: C_28_αββ(S); Peak 10: C_28_ααα(R); Peak 11: C_29_ααα(S); Peak 12: C_29_αββ(R); Peak 13: C_29_αββ(S); Peak 14: C_29_ααα(R)].

The total steranes in GB and DWH samples were found to be 221±5 and 720±30 mg/kg oil, respectively. Similar to hopane data, sterane data can also be used for source identification. Fig. 8 shows the chromatograms of steranes (at m/z 217) for both GB and DWH residues. The data show that steranes in GB residue are dominated by several high molecular weight compounds (such as C_29_-steranes). We have identified several sterane peaks based on published data [22,23] and used them to compute various diagnostic ratios that are suggested in the literature [14,18,19]; these results are summarized in Table 2. The sterane dataset provides an additional line of evidence for identifying and differentiating other residues from these two oil spills.

### Chemical Characterization Data for PAH Compounds

**Fig 9:**
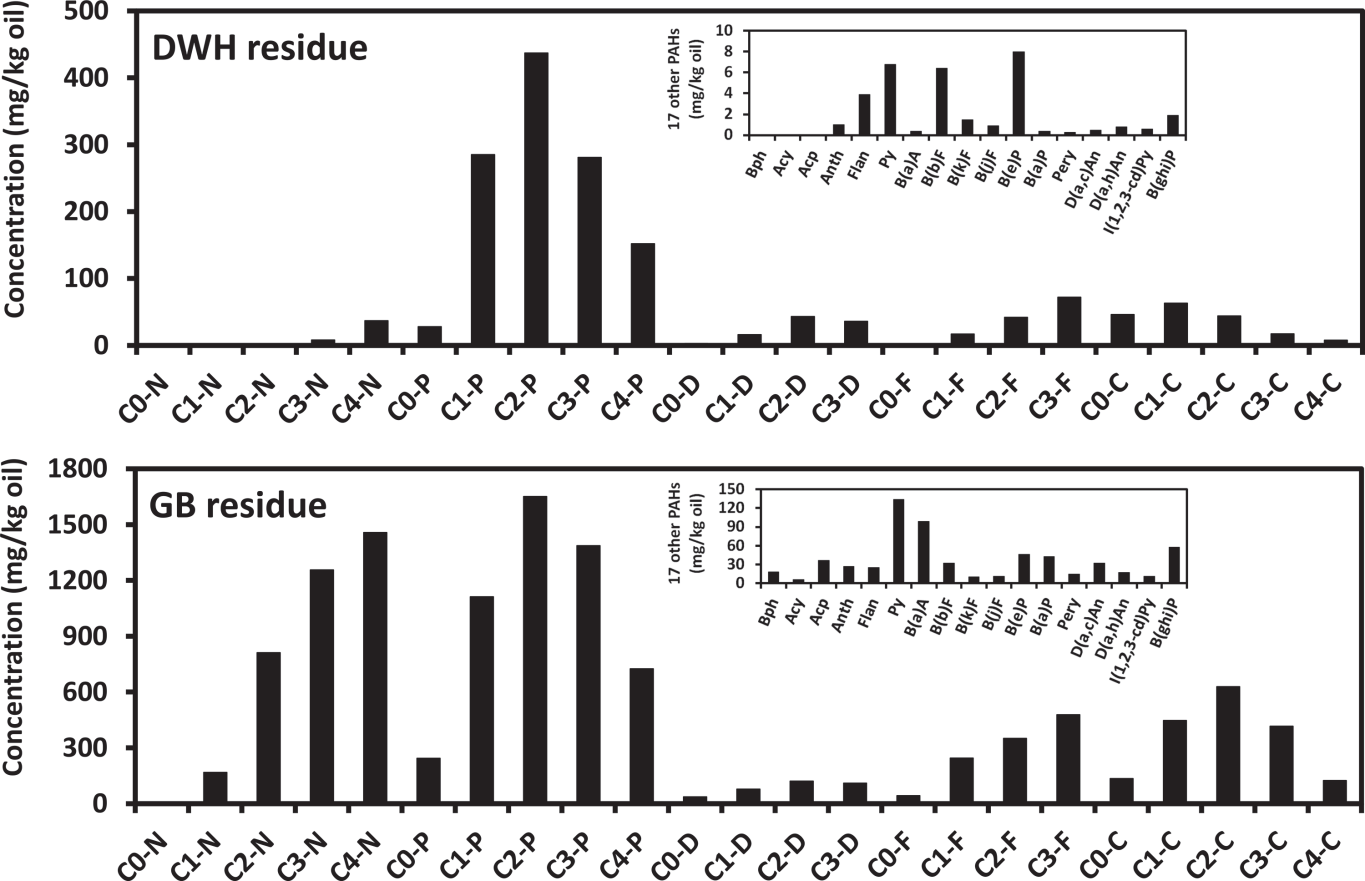
Concentration levels of various PAHs and alkylated PAH homologs measured in Deepwater Horizon and Galveston Bay oil spill residues.

Fig. 9 presents PAH concentration levels measured in DWH and GB residues. In Table 3 we summarize these concentrations in terms of five groups of alkylated PAHs (namely naphthalenes, phenanthrenes, dibenzothiophenes, fluorenes and chrysenes) with their parents, and seventeen other PAHs. The extracted ion chromatograms used for quantifying the alkylated PAHs in the GB residue are shown in S1 Chromatogram, S2 Chromatogram, S3 Chromatogram, S4 Chromatogram, S5 Chromatogram. For the DWH sample, the total amount of PAHs was estimated to be 1,714 mg/kg oil. The data also show that the five groups of alkylated PAHs were the most dominant compounds and they accounted for about 95% of total PAHs. Among the five groups, phenanthrenes (Group-2) were the most abundant compounds in the DWH sample with a total concentration of 1,183 mg/kg oil (which is about 69% of total PAHs), followed by chrysenes (Group-5) with a total concentration of 178 mg/kg oil (which is 10% of total PAHs), fluorenes (Group-4) with a total concentration of 132 mg/kg oil (which is 8% of total PAHs), dibenzothiophenes (Group-3) with a total concentration of 98 mg/kg oil (which is 6% of total PAHs), and naphthalenes (Group-1) with a total concentration of 46 mg/kg oil (which is 3% of total PAHs). The total concentration of all other 3- to 6-ring parent PAHs was estimated to be 33 mg/kg oil; biphenyl was not detected in the DWH sample.

**Table 3:** Summary of average PAH concentration levels measured in Deepwater Horizon and Galveston Bay oil spill residues (unit: mg/kg oil).

Compound	DWH residue [10]	GB residue
Five groups of alkylated PAHs and their parents		
Group-1: C_0_- to C_4_-naphthalenes	46	3,699
Group-2: C_0_- to C_4_-phenanthrenes	1,183	5,119
Group-3: C_0_- to C_3_-dibenzothiophenes	98	345
Group-4: C_0_- to C_3_-fluorenes	132	1,117
Group-5: C_0_- to C_4_-chrysenes	178	1,751
Sum of five groups of PAHs	1,636	12,031
Other seventeen PAHs		
Biphenyl (2-ring)	-	18
Sum of 3 to 6-ring PAHs	33	603
Total amount of PAHs	1,714	12,651

The total amount of PAHs measured in the GB sample was 12,651 mg/kg oil, which is about 7 times higher than the levels measured in the DWH sample (see Table 3). Similar to the DWH sample, PAHs in the GB sample were also dominated by the five groups of alkylated PAHs and accounted for about 95% of total PAHs. However, the relative distribution of various types of PAHs in the GB residue was different from the distribution observed for the DWH residue (see Fig. 9). More importantly, individual concentration levels of almost all the PAHs measured in the GB sample were much higher than the levels measured in the DWH sample. Interestingly, phenanthrenes are also the most abundant group of PAHs in GB residue and the total concentration of phenanthrenes (Group-2) estimated was 5,119 mg/kg oil (which is about 40% of total PAHs). This concentration level is about 4 times higher than the level measured in the DWH sample. The second dominant group of compounds are naphthalenes (Group-1) with a total concentration of 3,699 mg/kg oil (which accounted for about 29% of total PAHs); this level is about 80 times higher than the level observed in the DWH sample. The next group is chrysenes (Group-5) with a total concentration of 1,751 mg/kg oil (which is about 14% of total PAHs); followed by fluorenes (Group-4) with a total concentration of 1,117 mg/kg oil (which is about 9% of total PAHs); and dibenzothiophenes (Group-3) with a total concentration of 345 mg/kg oil (which accounted for about 3% of total PAHs). The concentration of biphenyl and the sum of other 3- to 6-ring PAHs were estimated to be 18 mg/kg oil and 603 mg/kg oil, respectively. Since GB residues were collected a short time after the spill, the concentration levels of light molecular weight PAHs, such as naphthalenes and biphenyl (volatile compounds that can easily evaporate during the early stage of a spill) were high, indicating the GB sample experienced very little weathering.

According to the Agency for Toxic Substances and Disease Registry [24], most heavy PAHs are either known or probable human carcinogens. For example, the 5-ring compound benzo[*a*]pyrene (BaP) has been shown to cause chromosomal replication errors, and can also affect human fertility levels. The concentration levels of BaP in GB and DWH samples were estimated to be 43 and 0.4 mg/kg oil, respectively. These data suggest that, in terms of BaP toxicity effects, GB residues will be substantially more toxic than DWH residues. Furthermore, concentrations of several alkylated PAHs in the GB residue were relatively high. Previous studies have shown that alkylated phenanthrenes, for example, can induce various types of ecological toxic effects in marine organisms [25–27]. Our data show that phenanthrene levels in the GB sample were approximately 4 times higher than the DWH sample, and these values were mostly dominated by alkylated phenanthrenes. These data also indicate that GB residues might be more toxic than DWH residues. Furthermore, recent studies have demonstrated that alkylated chrysenes in oil residues are likely to be recalcitrant for many years [10,28]. Emerging research has shown that although the measured aqueous solubility limits of individual alkylated chrysenes are very low, these chemicals could still induce chronic toxic effects in certain sensitive species, e.g., Japanese medaka embryos (*Oryzias latipes*) [29]. Additionally, studies have shown that the toxic effects of multiple PAHs present in oil spill residues can be additive [30]. Therefore, based on total PAHs alone, the much higher concentrations present in GB residues are likely to cause far more adverse effects to fishes and other marine species. However, since the toxicity of individual PAHs can vary significantly [31–34], better understanding of overall detrimental ecological effects associated with GB and DWH spills warrants further studies.

## Conclusions

This is the first study that reports field observations and chemical characterization data for the 2014 GB oil spill and compares the results against observations made during another major spill (the DWH oil spill). Our data document the differences in weathering patterns of GB and DWH oil spill residues. When compared to DWH first-arrival oil residue, the GB first-arrival sample experienced much less weathering due to the proximity of the accident site to the shoreline. Furthermore, the environmental weathering characteristics of the heavy, highly refined GB fuel oil are vastly different from the characteristics of raw light, sweet crude oil released during the DWH spill. For example, heavy fuel oil (like the GB oil) is expected to have a very low evaporation rate [35]. In comparison, as documented in previous studies, the evaporation rate of DWH raw crude oil is very high and evaporation process alone likely removed over 40% of the DWH oil mass within a week of surface weathering [10,36].

The hopane fingerprinting data show that GB residue has a wider m/z 191 chromatogram and displays a distinctly different fingerprint compared to the DWH fingerprint. Interestingly, both GB and DWH residues had similar amounts of total hopanes; however, the relative ratios of various types of hopane were different, yielding distinctly different fingerprints. GB residue also showed distinctly different sterane fingerprints from the DWH residue; also, the total amount of sterane measured in the GB residue was about three times lower than the DWH residue.

DWH oil spill residues arriving along GOM beaches interacted with suspended sand in nearshore waters and formed submerged oil mats (SOMs; often called tar mats). SOMs are continuously worked by waves and other nearshore processes yielding surface residual oil balls (SRBs; often called tar balls). Field studies have shown that the presence of SRBs continue to be a problem along Florida and Alabama beaches more than four years after the DWH spill, especially after major storm events [8,12,37]. Also, PAHs and their post-spill derivatives (such as oxygenated species whose effects are yet to be determined), trapped in buried DWH oil spill residues, continue to pose serious environmental concerns [16,38–42]. For the GB spill, early predictions indicated that the oil would be carried by northeasterly winds out into the GOM, and onshore winds would deposit oil onto various beaches [35]. Later, overflight observations documented beached oil being rapidly buried under clean sand on Matagorda Island, located about 200 km away from the spill location [1]. Based on both predicted and observed oil spill trajectories, and also based on the prior knowledge gained from studying the DWH accident, GB oil should have formed SRBs containing heavy fuel oil that are potentially distributed along various beaches located to the southwest of the GB. Since GB residues contain much higher levels of PAHs, these SRBs could pose long-term environmental risks. However, the total amount of oil released from the GB spill is substantially low when compared to the DWH spill and hence the spatial extent of these impacts will likely be small. Managing oil spill impacts to beach systems is a significant environmental challenge, and it becomes more complex in systems that experience multiple spill events (such as the GB system). The biomarker and PAH datasets provided in this study are important baseline information for monitoring the long-term fate and the potential environmental impacts of the GB oil spill event.

## Supporting Information

S1 Chromatogram Extracted ion chromatograms of alkylated naphthalene homologs in GB sample. (DOCX)

S2 Chromatogram Extracted ion chromatogram of alkylated phenanthrene homologs in GB sample. (DOCX)

S3 Chromatogram Extracted ion chromatogram of alkylated dibenzothiophene homologs in GB sample. (DOCX)

S4 Chromatogram Extracted ion chromatogram of alkylated fluorene homologs in GB sample. (DOCX)

S5 Chromatogram Extracted ion chromatogram of alkylated chrysene homologs in GB sample. (DOCX)
