# Correction: A Tale of Two Recent Spills—Comparison of 2014 Galveston Bay and 2010 Deepwater Horizon Oil Spill Residues

**DOI:** 10.1371/journal.pone.0124645

**Published:** 2015-04-08

**Authors:** Fang Yin, Joel S. Hayworth, T. Prabhakar Clement

Information is missing from the Funding section. The complete funding statement is: This work was supported, in part, by funding received from the Alabama Emergency Management Agency (AEMA). The GC/MS/MS facility and the characterization methods were developed using grant funds received from the National Science Foundation (NSFMRI grant #G00006697), Samuel Ginn College of Engineering, Auburn University, and Marine Environmental Sciences Consortium (MESC).

The Acknowledgments section is missing. The Acknowledgments should read: The logistic support provided by Dr. Janarthanan during our field survey is kindly acknowledged. We also like to thank the PLOS ONE reviewers and the Academic Editor Dr. James P. Meador for providing constructive review comments. All the authors have approved this final version and their relative responsibilities for this effort are: FY (55%), TPC (35%), and JSH (10%).
