# Supplementary figures and images for: A Tale of Two Recent Spills—Comparison of 2014 Galveston Bay and 2010 Deepwater Horizon Oil Spill Residues
Source: PLoS One. 2015 Feb 25;10(2):e0118098. doi: 10.1371/journal.pone.0118098 (PMC4340883; doi:10.1371/journal.pone.0118098)

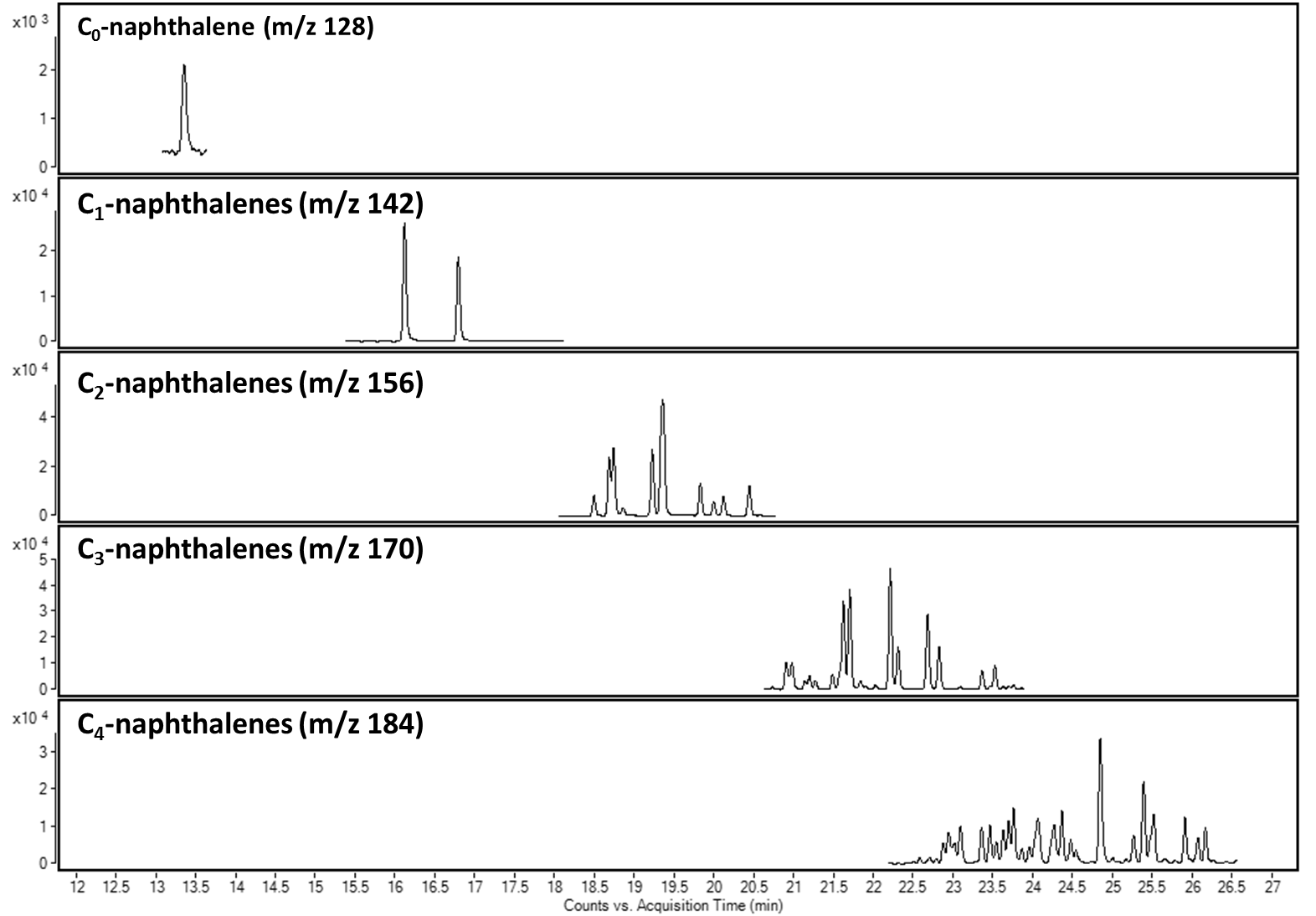


**S1. Extracted ion chromatograms of alkylated naphthalene homologs in GB sample**

Supplement: S1 Chromatogram — (DOCX) [file pone.0118098.s001.docx]

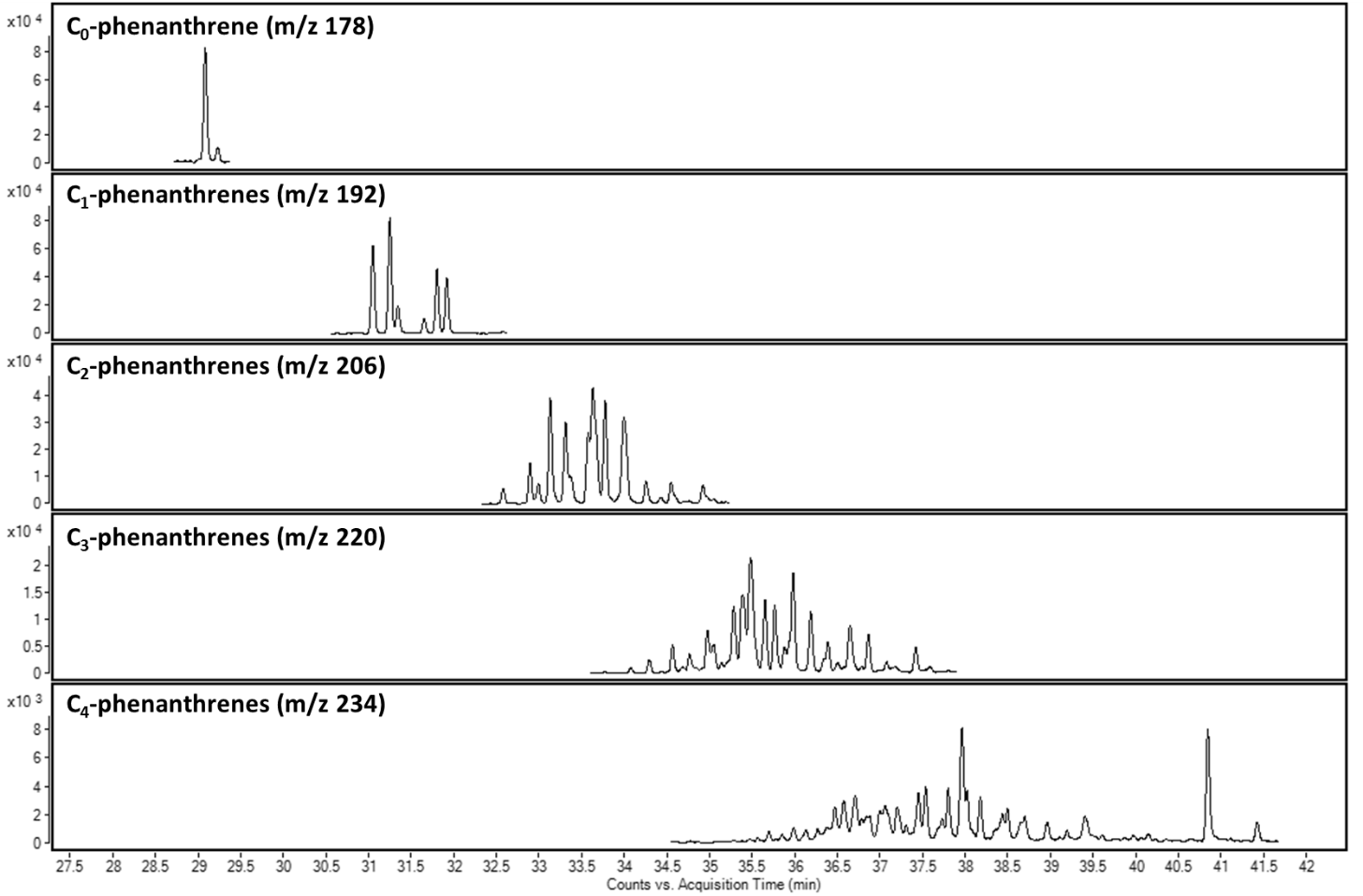


**S2. Extracted ion chromatogram of alkylated phenanthrene homologs in GB sample**

Supplement: S2 Chromatogram — (DOCX) [file pone.0118098.s002.docx]

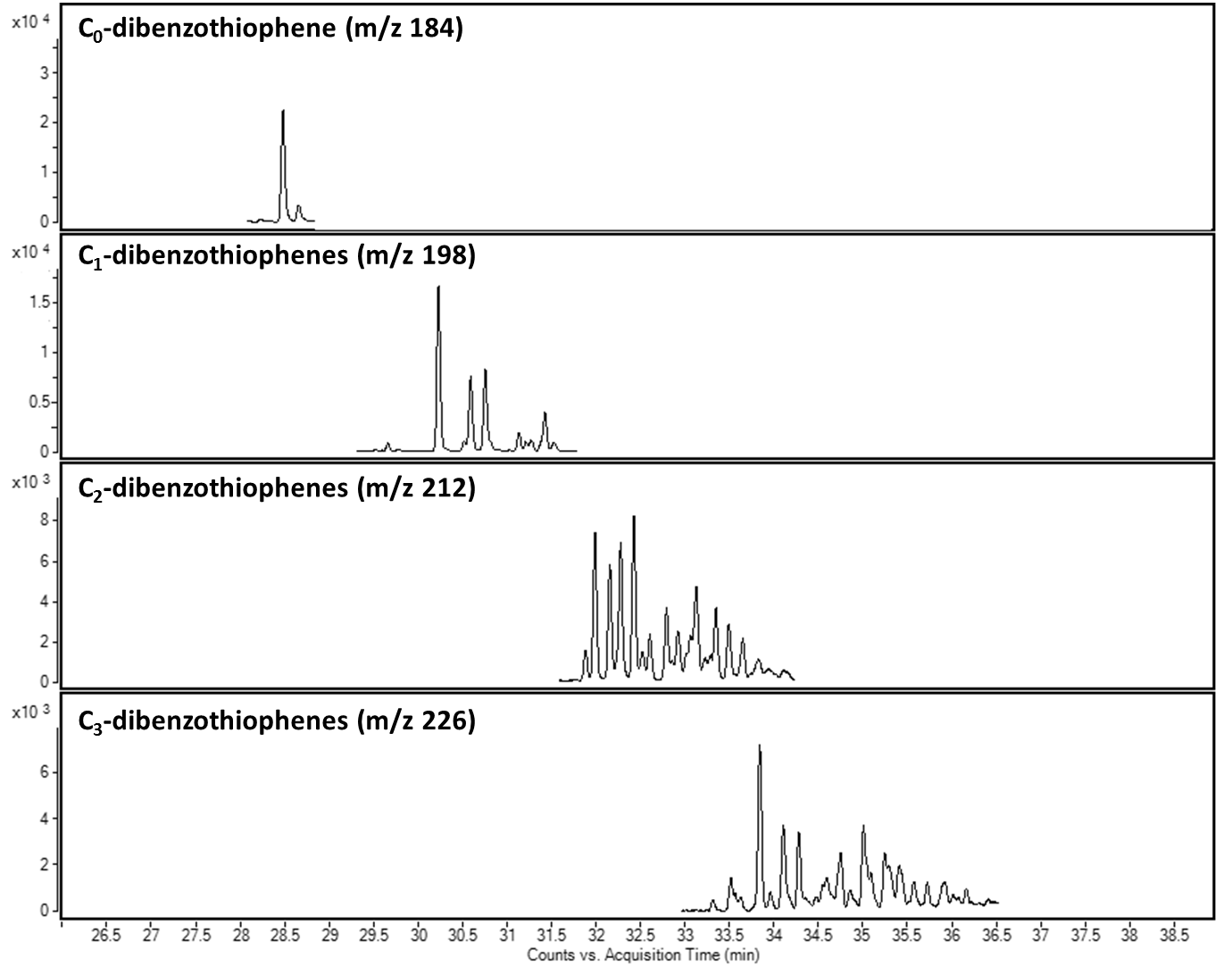


**S3. Extracted ion chromatogram of alkylated dibenzothiophene homologs in GB sample**

Supplement: S3 Chromatogram — (DOCX) [file pone.0118098.s003.docx]

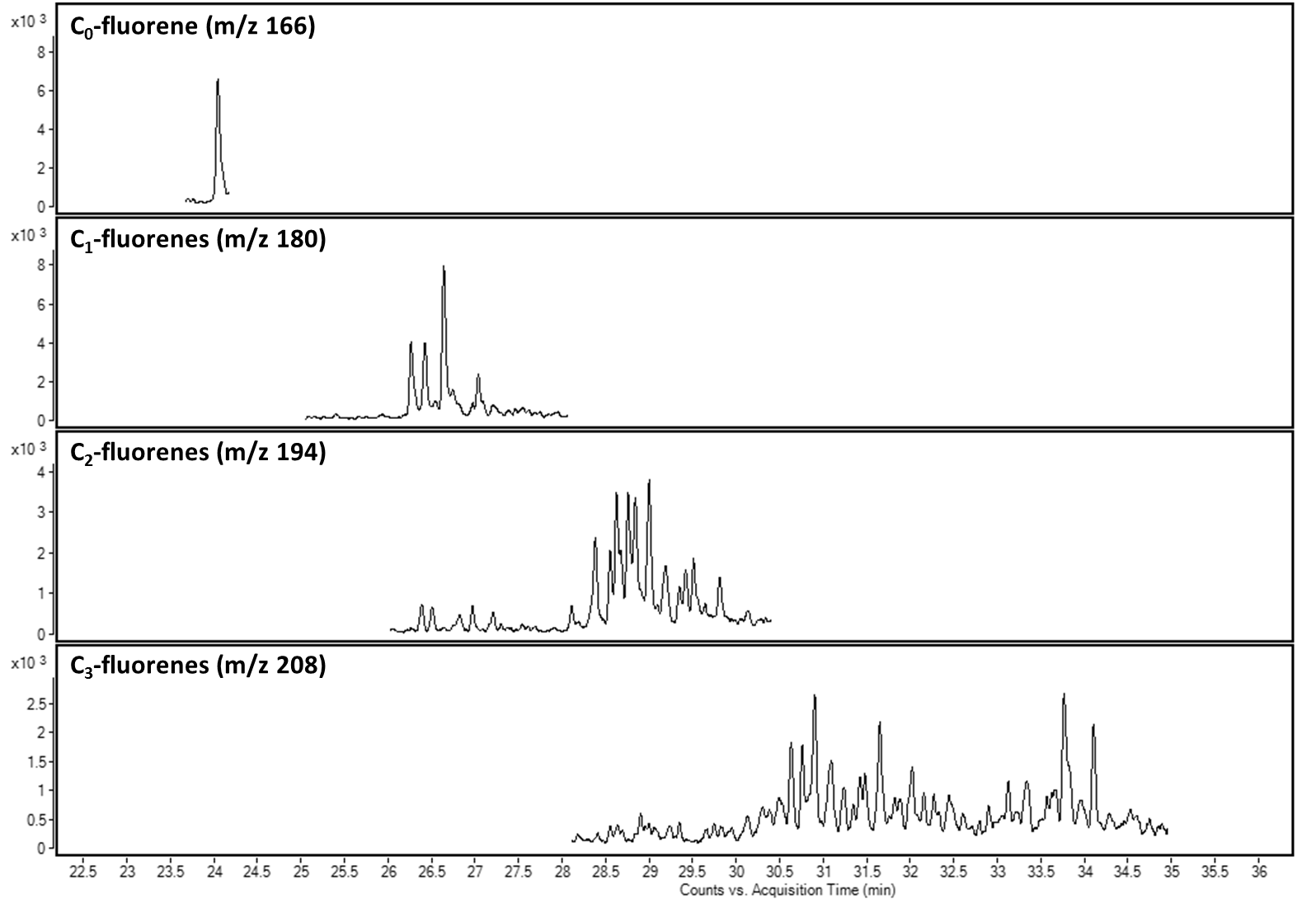


**S4. Extracted ion chromatogram of alkylated fluorene homologs in GB sample**

Supplement: S4 Chromatogram — (DOCX) [file pone.0118098.s004.docx]

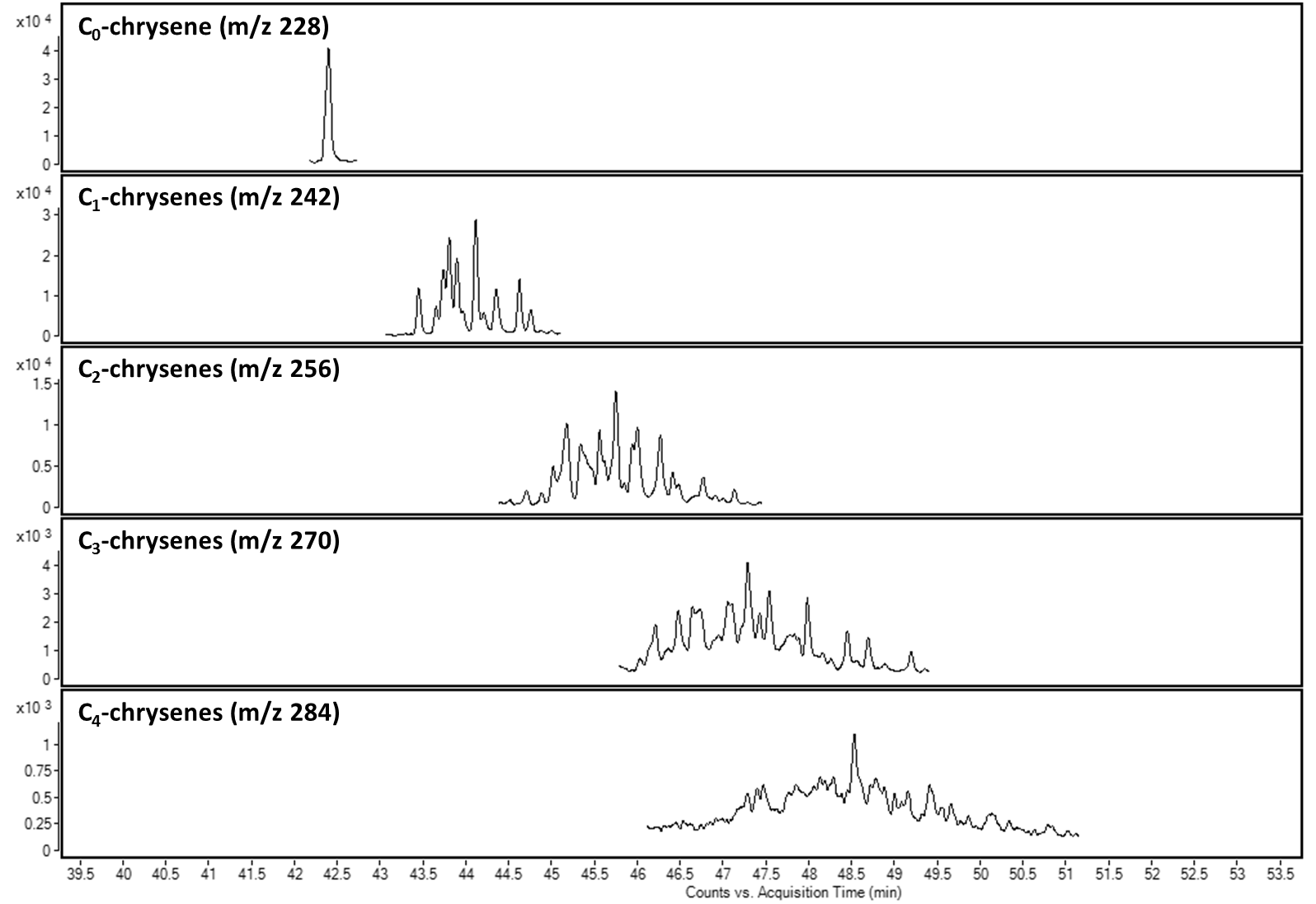


**S5. Extracted ion chromatogram of alkylated chrysene homologs in GB sample**

Supplement: S5 Chromatogram — (DOCX) [file pone.0118098.s005.docx]
